# Novel electrical properties and applications in kaleidoscopic graphene nanoribbons

**DOI:** 10.1039/d1ra05902e

**Published:** 2021-10-15

**Authors:** Wenjing Bo, Yi Zou, Jingang Wang

**Affiliations:** College of Science, Liaoning Petrochemical University Fushun 113001 China jingang_wang@lnpu.edu.cn happylele1989@126.com

## Abstract

As one of the representatives of nano-graphene materials, graphene nanoribbons (GNRs) have more novel electrical properties, highly adjustable electronic properties, and optoelectronic properties than graphene due to their diverse geometric structures and atomic precision configurations. The electrical properties and band gaps of GNRs depend on their width, length, boundary configuration and other elemental doping, *etc.* With the improvement of the preparation technology and level of GNRs with atomic precision, increasing number of GNRs with different configurations are being prepared. They all show novel electrical properties and high tunability, which provides a broad prospect for the application of GNRs in the field of microelectronics. Here, we summarize the latest GNR-based achievements in recent years and summarize the latest electrical properties and potential applications of GNRs.

## Abstract 2

For quasi-one-dimensional graphene nanoribbons (GNRs), adjusting its length, width, doping and heteroatom adsorption showed novel electronic properties. He has a very wide range of potential applications in the field of microelectronics.

## Graphical abstract

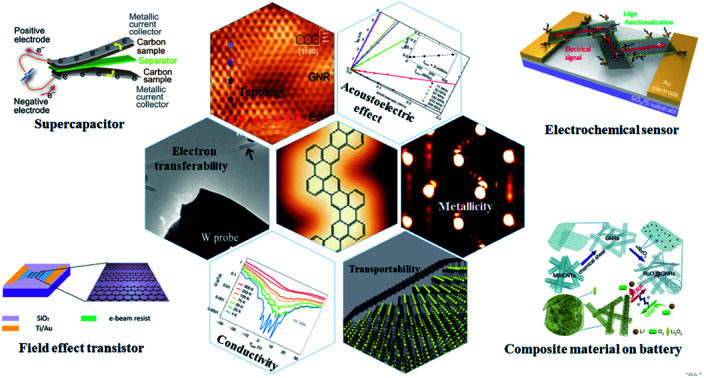

## 1 Introduce of GNRs

Since graphene nanomaterials were prepared in 2004, ribbon-shaped graphene nanomaterials have been studied. Studies have shown that GNRs are mainly sheared from two-dimensional graphene or carbon nanotubes. According to different boundary configurations, they are divided into the zigzag boundary (z-GNRs) and armchair boundary (a-GNRs). Based on the size effect of nanomaterials, GNRs with different boundary configurations show very different physical and chemical properties. Since GNRs are cut through graphene, the carbon atoms at the edges of GNRs are in an unsaturated state. This active edge state has become a very important factor in determining the edge structure. For a-GNRs, the two-dimensional planar structure of graphene is basically maintained, but for z-GNRs, the edge structure will be reconstructed at higher temperatures. In recent years, a variety of new two-dimensional semiconductor materials have shown good light responsiveness. Some nanosheets can be used as building blocks for various optoelectronic devices. These provide new research ideas in the field of future quantum communications, optoelectronic applications and other photodetectors.^1–3^

### 1.1 The lattice structures of GNRs

When discussing the characteristics of microscopic materials such as GNRs, many tools are used: such as Raman spectroscopy, scanning electron microscopy and transmission electron microscopy. When GNRs have different shapes, their Raman activity is different. In Raman spectroscopy, the chemical bonds, functional groups, number of layers, doping, defects and edge conditions of GNRs can be identified, and basic problems such as electrochemical interface structure, adsorption and reaction can be studied in depth. Electron microscopy techniques are also crucial, such as scanning electron microscopy (SEM) and transmission electron microscopy (TEM). SEM is used to depict the surface molecular structure at the nanometer level. Sometimes when facing ultra-thin characteristic materials that are easily scattered by electrons or absorbed by objects, the penetrating power is low, so TEM is used for observation. These advantageous tools provide great help to control the accuracy of GNRs.^4–7^

GNRs obtained by etching graphene can exhibit arbitrary shapes. Although the etching method is simple to perform, the edge structure is not clear enough and cannot be prepared on a large scale. Therefore, the bottom-up method of preparing GNRs came into being. In recent years, surface synthesis technology has developed rapidly. It can precisely control the edge structure of GNRs prepared with atomic-level precision and can produce GNRs on a large scale by chemical vapor deposition. Due to the high activity and instability of the edge structure of the nanoribbons, the length of the synthesized GNRs is limited. But the nanobelt synthesized in solution overcomes this shortcoming. At present, the longest GNRs are obtained using in-solution synthesis methods. With theoretical calculations and experimental studies, studying new physical properties and applications of GNRs are becoming research hotspots.^8–37^

**Fig. 1:**
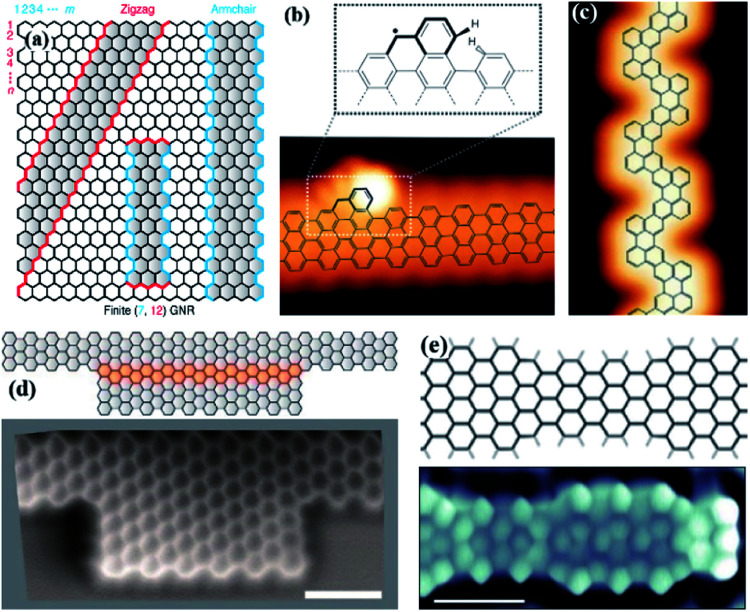
(a) Schematic of the structure of GNRs with zigzag borders (red) and armchair-shaped borders (blue). Indices are used to denote the dimensions of GNRs along the zigzag (*m*) and armchair direction (*n*), respectively.^38^ (b) STM images corresponding edge extensions overlapping the structural model 7-a-GNRs. Scale bar, 1 nm.^39^ (c) STM topography of sn-8-a-GNRs.^40^ (d) Schematic of 14-a-GNRs quantum dots formed by the edge fusion of two 7-a-GNRs, and its non-contact atomic force microscopy (nc-AFM) image.^41^ (e) The local topological electronic state of GNRs and a micrograph of one end of the nanoribbons. Scale bar, 1 nm.^42^

According to the direction shown, z-GNRs and a-GNRs can be cut out (Fig. 1(a)).^38^ 7-a-GNRs with a single edge extension (structure highlighted by the dashed rectangle) (Fig. 1(b)).^39^ Sun's research team synthesized staggered narrow *N* = 8 a-GNRs (sn-8-a-GNRs) through a bottom-up approach, with alternating “bite” defects on the other side (Fig. 1(c)).^40^ Two 7-a-GNRs are fused to form 14-a-GNRs quantum dots by cross-dihydrogen coupling lateral edge fusion. Its image can be seen under non-contact atomic force microscopy (NC-AFM) (Fig. 1(d)).^41^ Researchers have synthesized GNRs (black), a two-dimensional form of carbon. GNRs have narrow parts (white) and wide parts (blue) alternating with each other. The photomicrograph shows one end of the nanoribbon (Fig. 1(e)).^42^

### 1.2 The electronic structure of GNRs

Graphene is a semi-metallic material with a zero band gap. Adjusting the band gap of graphene nanoribbons is a way to open up the properties of GNRs. For example, a-GNRs with a small length has a larger band gap, when both the length and width are small, the band gap of z-GNRs is larger, the double-layer structure has a different band gap than the single-layer structure; doping and adsorption can also control the material structure, *etc.*^43–57^ From previous studies, we know that a-GNRs with uniform jagged edges have an energy gap, and the energy gap decreases as the width of the nanoribbon increases. The edge effect is crucial for determining the value of the band gap and the rule of proportionality. The ideal quasi-one-dimensional (1D) quantum confinement is significantly functionalized, which proves that the growth of graphene leads to the formation of silver nanoribbons of different widths and exhibits semiconductor or metallic behavior.^58–67^ The synthesis method for doping of heteroatom on the surface of GNRs, which is a modulation strategy for GNRs with special electronic properties.

**Fig. 2:**
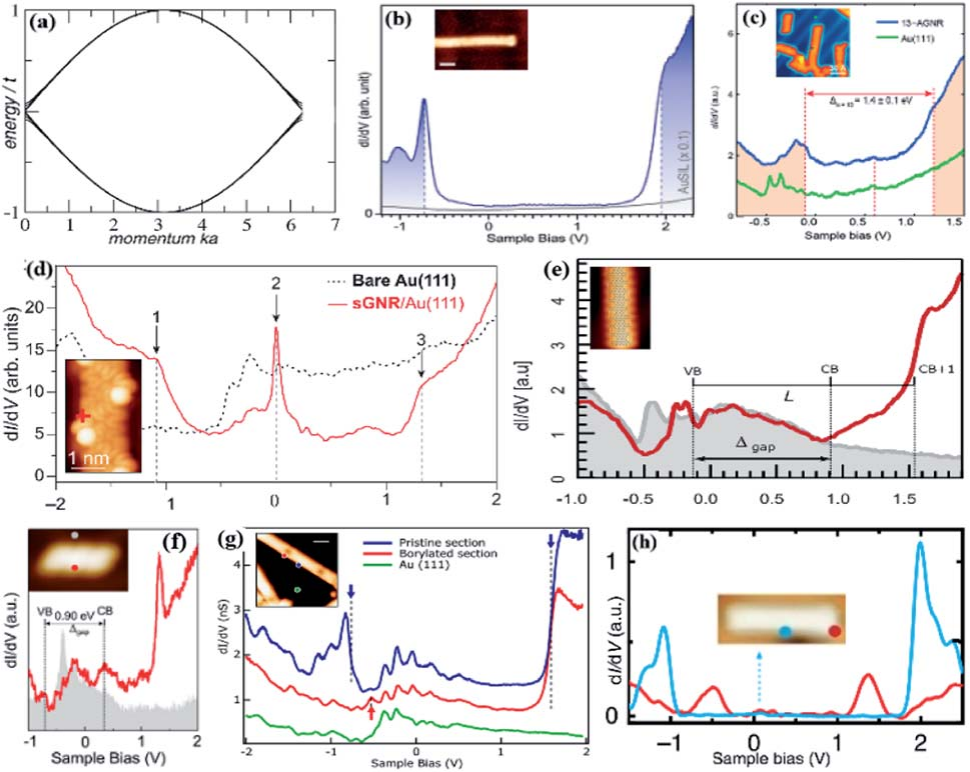
(a) Electron dispersion energy spectrum of GNRs.^68^ (b) d*I*/d*V* spectrum of 7-a-GNRs. The inset shows that Si intercalates the STM image of 7-a-GNRs.^69^ (c) d*I*/d*V* spectra of 13-a-GNRs band gap measured by STM (blue line).^70^ (d) STM image of a segment of sawtooth-GNRs (s-GNRs). d*I*/d*V* point spectroscopy of s-GNRs/Au(111) at the zigzag position marked in the inset. The dashed curve shows the bare Au(111) reference spectrum.^71^ (e) d*I*/d*V* spectrum acquired at the multibay edge of a 7–13-a-GNRs.^72^ (f) d*I*/d*V* spectra taken on nitrogen–boron–nitrogen (NBN)-z-GNRs.^73^ (g) d*I*/d*V* spectra of the mixed GNRs obtained at different locations.^74^ (h) d*I*/d*V* spectra of (7,20) GNRs. Inset: STM image under a bias voltage corresponding to the band gap sample.^38^

The energy spectra of 14 eigenstate nanoribbons with armchair edges are calculated according to the tight-binding equation (Fig. 2(a)). The width of the nanoribbons is *N* = 200 units.^68^ The STM image after depositing Si on 475 K substrate and inserting 1 ml of the Si incorporated Au(111) (AuSIL). The differential conductance (d*I*/d*V*) spectra recorded on AuSIL show the boundary state of 7-a-GNRs (Fig. 2(b)).^69^ By performing scanning tunneling spectroscopy (STS5) measurements, the local electronic structure of 13-a-GNRs was determined. The blue line represents the characteristic d*I*/d*V* spectra of 13-a-GNRs (Fig. 2(c)).^70^ The red spectra were acquired at the edge of the borylated section, the green spectra were obtained on Au(111) substrate. The blue arrow and dotted line mark the beginning of the original and borylated part of the conduction band (CB) and the beginning of the original segment of the valence band (VB) (Fig. 2(d)).^71^ d*I*/d*V* spectra obtained at the multi-interval edge of 7–13-a-GNRs can identify the beginning of each band (Fig. 2(e)).^72^ In d*I*/d*V* spectra, CB is 0.20 eV and VB is 0.70 eV, so the electronic band gap is 0.90 eV. This is consistent with density functional theory (DFT) calculations (Fig. 2(f)).^73^ By performing STS measurements at different locations, it is possible to discover the influence of boron on the embedded electronic structure of the GNRs (Fig. 2(g)).^74^ The STM scan image of (7, 20) GNRs on NaCl, and d*I*/d*V* spectra exhibit two peaks centered at −0.5 V and 1.3 V (Fig. 2(h)).^38^

To better understand the electronic properties of GNRs, researchers use d*I*/d*V* spectra for detection. The d*I*/d*V* spectra reflect as much as possible the local electronic density of states at a certain energy in real space. According to the width and edge geometry of CNRs, it provides a viable strategy for GNRs with jagged edges and adjustable electronic properties.^75–82^

## 2 Electrical properties of GNRs

### 2.1 Metallicity in GNRs

It is challenging to design and manufacture strong metallic states in GNRs because when graphene is patterned at the nanoscale, lateral quantum confinement and multi-electron interactions can cause electronic band gaps. The bottom-up synthesis method has the latest development, which makes it possible to design and characterize atomically accurate GNRs, but the strategy to achieve GNRs metallicity has been elusive.^83–96^

**Fig. 3:**
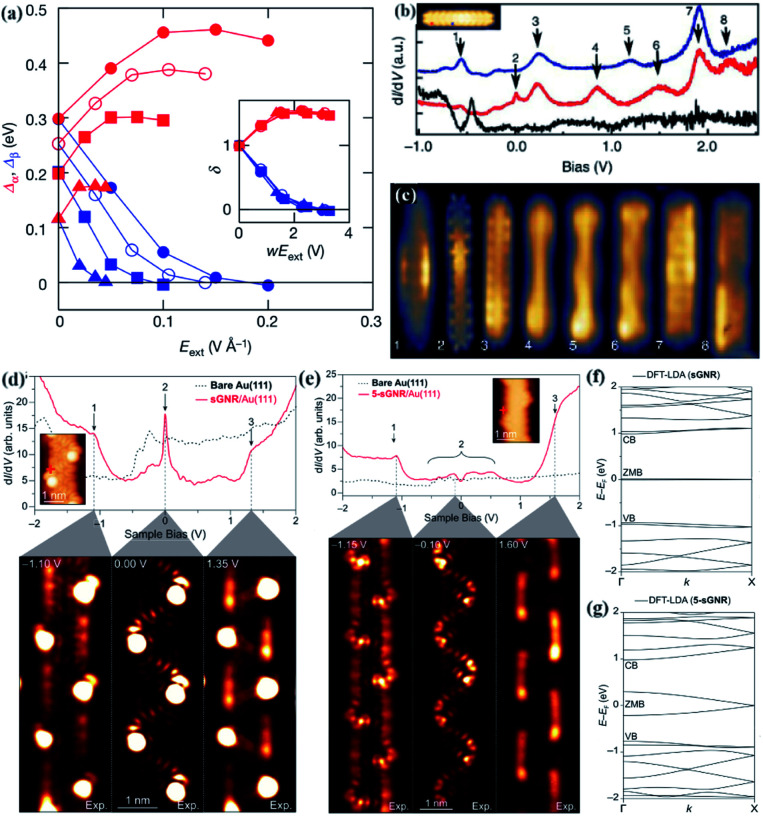
(a) The direct band gap (red line) and indirect band gap (blue line) of GNRs at different widths.^45^ (b) 5-Monomer long GNRs of d*I*/d*V* spectra with a metallic tip.^97^ (c) Experimental constant-height d*I*/d*V* spectra.^97^ (d) Constant-height d*I*/d*V* spectra of s-GNRs conducted at the biases.^71^ (e) Constant-height d*I*/d*V* maps of 5-s-GNRs (right) conducted at the biases.^71^ (f) Local Density Approximation of Density Functional Theory (DFT-LDA) calculated band structure for (d).^71^ (g) DFT-LDA calculated band structure for (e). The valence, zero-mode, and conduction bands are labeled VB, ZMB, and CB, respectively.^71^

The half-metal degree is dependent on the system size, different symbols represent z-GNRs of different lengths. When changing the direction of the external electric fields (*E*_ext_), the spin polarity of the carriers in the half-metal strip will also be reversed, because the induced potential at the edge changes its sign. When the critical electric field decreases as the width increases, z-GNRs exhibit half-metallicity. Because at this time the electrostatic potential difference between the two edges is proportional to the system size (Fig. 3(a)). If a transverse electric field is applied to the zigzag edge of graphene nanoribbons, its semi-metallic properties are also stable, which provides effective help for exploring graphene-based nanoscale spintronics.^45^ d*I*/d*V* spectra of 5-monomer GNR at different positions. In addition, there are corresponding experimental and calculated conductance diagrams (Fig. 3(b) and (c)).^97^ The occupied states are at −550 mV and 26 mV. Although all GNRs experimental studies have shown a very wide band gap. They studied that GNRs with a length of 5 nm has reached almost metallic performance at a band gap of about 100 meV. The metal abundance of GNRs was determined experimentally, and its electronic structure was characterized through STM images. The typical d*I*/d*V* point spectra were obtained on s-GNRs. A serpentine pattern is observed near the zero-mode band (ZMB) at the valence band edge (*V* = 0), indicating that the metal state seen in 5-s-GNRs and s-GNRs is very similar (Fig. 3(d)–(g)). This research also provides a useful tool for controlling the metallicity of GNRs and adjusting the electronic structure of GNRs to different physical states. At the same time, it also provides a way for the development of nano-scale equipment and electronic and magnetic phenomena in such one-dimensional metal systems, breaking through the metallic control technology of graphene nanoribbons.^71^

It is possible to adjust the metal abundance in GNRs through the methods described above, inserting the symmetrical superlattice of the zero-energy mode into other semiconductor GNR methods, bottom-up synthesis methods, and so on.

### 2.2 Topological properties in GNRs

**Fig. 4:**
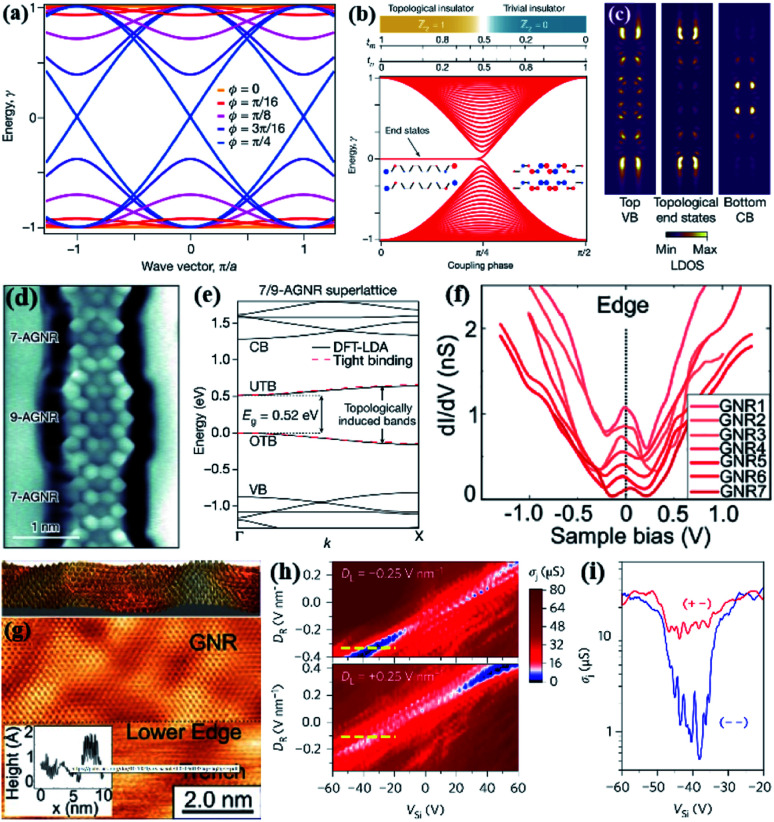
(a) Implementing the SSH model in GNRs.^98^ (b) Surface chemical synthesis method results in atomically accurate GNRs.^98^ (c) Tight-binding-simulated charge-density maps plot of VB top, *E* = 0 eV, and CB bottom.^98^ (d) A bond-resolved STM image of 7–9-a-GNRs superlattice shows the bond-resolved structure of the heterojunction interface.^42^ (e) DFT-LDA energy band structure of 7–9-a-GNRs superlattice.^42^ (f) Various STS spectra taken on the edge of the z-GNRs from seven different ribbons.^100^ (g) High-resolution STM image of the edge of the z-GNRs (+1.9 V, 0.1 nA).^100^ (h) The junction conductance *σ*_j_ at a function of *V*_Si_ at fixed values of *D*_R_ from *D*_R_ from −0.4 nm^−1^ to 0.4 V nm^−1^.^101^ (i) *σ*_j_*versus V*_Si_ along the yellow dashed lines marked in the upper (blue curve) and lower (red curve) panels.^101^

The orange corresponding band *E*(*k*,*φ*) is a non-dispersive band structure with two insulated chain configurations *φ* = 0 and *φ* = π/2, π/4. The band (blue) is a gapless metallic phase (Fig. 4(a)).^98^ The researchers conducted experiments under high vacuum conditions and deposited on the surface of Au(111) single crystals with an atomic-scale precision method. The synthesis of GNRs with alternating widths (Fig. 4(b)).^98^ The tunnel differential spectroscopy technique is scanned (Fig. 4(c)).^98^ The topological properties of GNRs are determined, which is a topological band structure that can be adjusted artificially. This has helped to achieve a one-dimensional material bandgap engineering, physics research is also a one-dimensional quantum spin contribution. The research team used molecular precursors to assemble experiments and obtained atomic-scale GNRs, which is consistent with the predicted valence electron structure in the Su–Schrieffer–Heeger (SSH) theoretical model (Fig. 4(c) and (e)). At the same time, it was found that there is a controllable periodic coupling in the topological boundary state at the junction of the GNRs.^42^ Another researcher discovered that a peak was found at the Fermi energy (*E*_F_), which is called a 1D topological surface state. This state may provide ballistic and spin-polarized transport channels (Fig. 4(f) and (g)).^99^ The existence of the kink state in the (positive and negative) electric field configuration (red curve) results in high conductance inside the band gap, while the blue curve *σ*_j_ is in the negative electric field configuration. The conductivity is low (Fig. 4(h) and (i)). This research uses electronic control to create a 1D topological conductive channel in bilayer graphene (BLG). By combining a narrower junction with a larger band gap, allows the kink valley electronic device to work at non-low temperature. This structure makes it possible to realize potential-controlled quantum dots with few electrons and opens the door to the edge states and the domain wall physics of the BLG quantum Hall system.^100^

The topology of GNRs can be further modified by dopants. The GNRs topological insulators are designed by experimental methods, and they have achieved strange topological states. The theoretical calculations are consistent with the experimental results. This precise regulation of the energy band of 1D materials based on electronic topology lays the foundation for the development of quantum computing technology in the future.^101–121^

### 2.3 Transport characteristics of GNRs

**Fig. 5:**
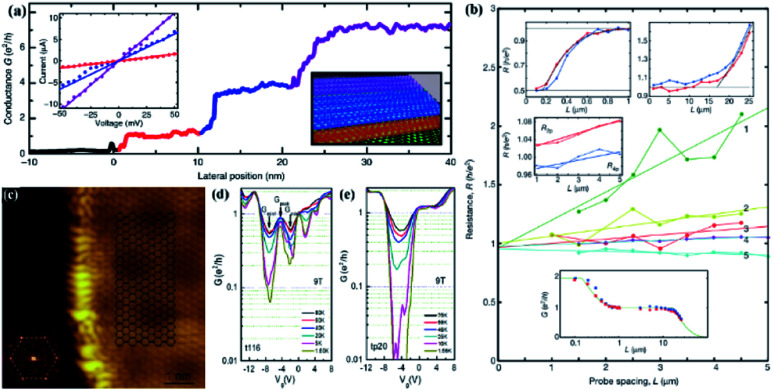
(a) Conductance measured for a fixed distance *L* = 70 nm. Inset: *I*–*V*-curves measured at of the GNRs.^122^ (b) Resistance *versus* probe spacing *L*.^123^ (c) STM image of the etched graphene.^124^ (d) Temperature-variable transport measurement results of z-GNRs under a 9 T magnetic field.^124^ (e) Temperature-variable transport measurement results of disordered edge GNRs under a 9 T magnetic field.^124^

Johannes *et al.* have studied a variety of GNRs and discovered their special transport properties. When studying the asymmetric sidewall nanoribbons between the upper and lower edges caused by the edge morphology, it was found that a unique isolated transport channel can be produced. When the moving tip is connected to the lower edge of the belt (Fig. 5(a)),^122^ the conductance is *e*^2^/*h*. When the tip moves from the edge to the whole, two platforms with higher conductivity will appear, the values of which are close to 4*e*^2^/*h* indicating transmission through an additional 4 times degenerate ballistic channel. A group of researchers presented strong evidence for a single-track ballistic transmission in long epitaxial GNRs (Fig. 5(b)).^123^ This means that both spin and valley degeneracies have increased. On the other hand, the conductance step of 4*e*^2^/*h* implies that the transport of sub-bands was induced by spin generation and valley degeneracy constraints, such as those expected in the original zigzag nanoribbons.

When the team conducted research on the z-GNRs, they discovered that the z-GNRs of different widths were processed on hexagonal boron nitride (h-BN) insulating substrates. Under the combined action of the strong magnetic field and the size effect of the nanoribbons, the “edge states” with a fill factor of zero (*V* = 0) were successfully insulated. A conductance peak related to the edge state was observed, which was not changing with temperature and magnetic field of robustness. At the same time, through non-local measurements, a voltage signal was also observed under zero magnetic field, and its energy position was consistent with the conductance peak under magnetic transport, further confirming that the edge states are involved in conduction. In addition, in the comparative samples of GNRs with disordered edges, this edge transport feature did not appear, which confirms that the edge conductivity is unique to the zigzag edge (Fig. 5(c)–(e)).^124^

The above series of interactions can provide new ideas for future development, including interfaces with lateral heterostructures, systems with emergent topological effects, and quantum Hall effects. Regardless of the source of the materials, in the future electronic products of GNRs, ballistic transport at room temperature will play a vital role.^125–142^

### 2.4 Electrical conductivity properties of GNRs

**Fig. 6:**
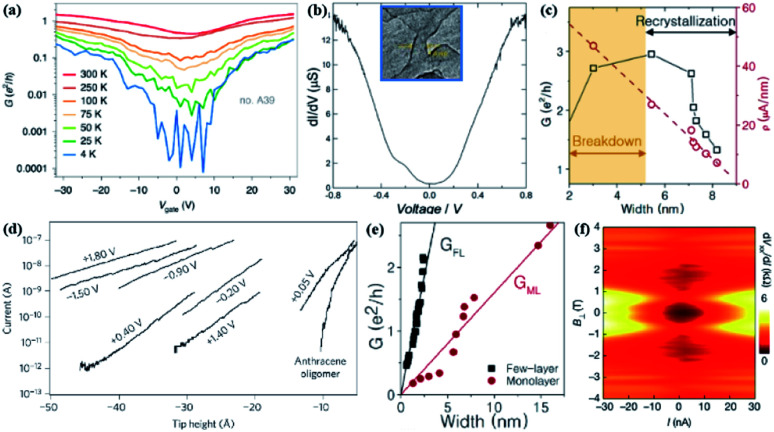
(a) Graph of the relationship between conductivity (*G*) and the back gate voltage (*V*_gate_) of the ∼5 nm wide a-GNRs device at different temperatures.^157^ (b) d*I*/d*V* as a function of bias voltage. Inset: TEM image of 1.6 nm wide graphene.^158^ (c) The conductance of GNRs when the Joule heating value increases.^159^ (d) Measure the conductivity diagram of a single molecule.^160^ (e) Conductivity of GNRs with different layers.^161^ (f) d*I*/d*V* spectra as a function of DC bias current and vertical magnetic field.^162^

The conductivity between the feature is used to study the interaction of atomic structure and electronic properties of GNRs. This kind of prospect will provide help in new cross-two-dimensional materials in the future.^143–156^ Transport measurements show that the band gap size of sub-7-nm-wide z-GNRs is inversely proportional to its width, while the band gap–width relationship of narrower a-GNRs exhibits volatility. Obvious conductance peaks are observed in the 8–10 nm wide z-GNRs transmission curve (Fig. 6(a)), but not in most of a-GNRs. At the same time, magnetic transport studies have shown that z-GNRs exhibit lower permeability, but a-GNRs have higher permeability. The successful preparation of h-BN surface embedded chirality-controllable GNRs provides a new way for complex nano-integrated circuits.^157^ The GNRs prepared by Joule heating can obtain GNRs with a width of about 10 nm. During the reduction process, the conductance nonlinearity of GNRs was displayed as a function of width (Fig. 6(b)).^158^

This proves that the known structure of GNRs, the opening and conductance differential characteristics of the band gap exists. They obtained the thinnest GNRS structure, with a width of about 1.6 nm, and 400 meV is observed by transportation measurement. The inherent electronic properties of GNRs can be studied in this way. The research team performed spatial mapping of the electronic structure of the long and narrow GNRs adsorbed on the Au(111) surface (Fig. 6(c)).^159^ This study provides an in-depth understanding of the reconstruction mechanism of a single nanoscale device detected *in situ*. In the sub-10 nm-wide GNRs device, lattice disorder and bonded double-layer edges are observed immediately after the pattern is formed. As the Joule heating increases, the GNRs continue to recrystallize while maintaining the combined double-layer edge. Although the width becomes narrower, its inherent conductivity is increased. It was found that the device width was reduced by nearly 3 times, the intrinsic conductivity doubled during the recrystallization process to 2.7*e*^2^/*h*, which indicated the limitation of the traditional pattern/etching process and the potential of Joule-heat recrystallization.

Some studies have discovered the electrical conductivity properties of single GNRs and linked them to the atomic structure and electronic state. When systematically measuring the stretching curves of different bias voltages (*i.e.*, the electrode electron energy relative to the movement of the GNRs), different slopes can be obtained (Fig. 6(d)),^160^ and each bias voltage has a characteristic attenuation constant. It is found that when the bias value increases, the slope of the conductance will decrease, so the conductance will increase. This proves the importance of the edge state and plane geometry. The researchers made controllable single-layer and few-layer GNRs with a width of less than 15 nm (Fig. 6(e)). The conductivity of double-layer GNRs is about *GFL*(*w*) ≈ 0.75(*e*^2^/*h*)*w*, the conductivity of single-layer GNRs is about 5 times that of the single-layer. The high conductivity of the few-layer GNRs is due to the bonding edge, which provides a stable structure and additional conduction channels. Single-layer GNRs form the armchair end edge after the current annealing, providing a way to prepare edge-specific GNRs.^161^ In experiments, it was found that the rhombohedral stacked trilayer graphene (ABC-TLG) and h-BN superlattice showed a Mott insulation state below 20 K. It is expected that the electronic behavior in the ABC-TLG/h-BN superlattice will very much depend on the interaction between the electronic interaction and the microstrip bandwidth. The magnetic field obviously inhibits the apparent superconductivity, and the superconductivity almost disappears, showing a wealth of adjustable behavior (Fig. 6(f)).^162^

People expect to effectively control the conductivity of these systems through the edge structure, whether in the bottom-up surface polymerization process or through the substrate. These developments will open up new directions for the band gap engineering of the next generation of nanoelectronics.

### 2.5 Other electrical properties of GNRs

**Fig. 7:**
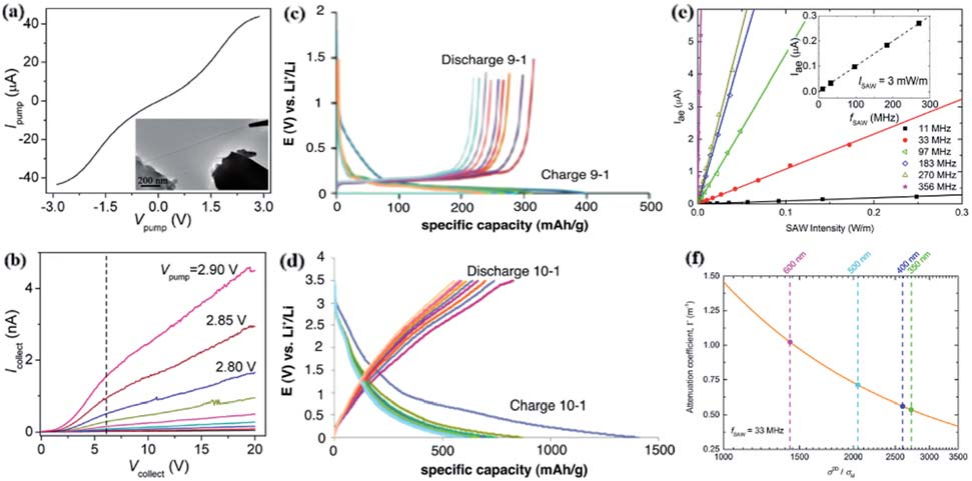
(a) Two-terminal transmission characteristic curve.^163^ (b) Different GNR curve measurements under different driving voltages.^163^ (c) The charge and discharge curve of medium carbon microbeads (MCMB) graphite.^164^ (d) Charge and discharge curve of ox-GNRs.^164^ (e) Acoustic current at several surface acoustic waves (SAW) frequencies.^165^ (f) Calculation diagram of attenuation coefficient.^165^

Wei *et al.* studied the first single GNR electron emission. Through the typical electron transfer characteristics of GNRs, when the driving voltage is 3 V, high emission density can be achieved (Fig. 7(a) and (b)).^163^ Electrons are driven out of a single GNR, and the emission current increases exponentially with the driving voltage. A group of research teams obtained the electrochemical lithium absorption capacity of carbonaceous 1D GNRs by decompressing the original multi-walled carbon nanotubes (MWCNTs). Studies have found that oxidized GNRs (ox-GNRs) are superior to other materials in terms of energy density. The reversible and irreversible capacities increase of ox-GNRs indicates that the presence of oxygen in GNRs induces more stable formation, chemically bound solid electrolyte interface (SEI) (Fig. 7(c) and (d)).^164^ This powerful SEI rich in Li elements may prevent electrode degradation and enhance the storage capacity of the electrode. The reversible capacity of ox-GNRs is in the range of 800 mA h g^−1^, the capacity loss per cycle in the early stage is about 3%, and the loss rate in the subsequent cycles gradually decreases.

The researchers studied the acoustoelectric effect in GNRs and showed that at room temperature, GNRs with a physical width as small as 200 nm can generate acoustic currents. In an 300 μm × 3 mm array composed of 500 nm wide GNRs, when the surface acoustic wave frequency is 442 MHz, the measurement found an acoustic current of as high as ∼5.5 μA (Fig. 7(e)).^165^ It is found that the acoustic current attenuation coefficient increases as the width decreases and the conductivity increases as the bandwidth decreases from 600 nm to 350 nm (Fig. 7(f)).^165^ From the induced acoustic current density equation, it is concluded that as the bandwidth decreases, not only the conductivity increases but also the mobility in the band. Because, during the preparation process, the increase in doping along the rough edges of the damage causes an increase in the acoustic current.

In just a few years, the development of GNRs has become a hot spot among the scientific community. We believe that these discoveries of electrical properties, acoustoelectric effects, electron emission, electrochemistry, acoustoelectric effects, electrocatalytic activity and other properties will eventually be widely used in various researches.^166–169^

## 3 Applications based on GNRs

### 3.1 The field-effect transistor (FET)

A semiconductor device can control the output loop current by controlling the electric field effect of the input loop, FET.^166–184^

**Fig. 8:**
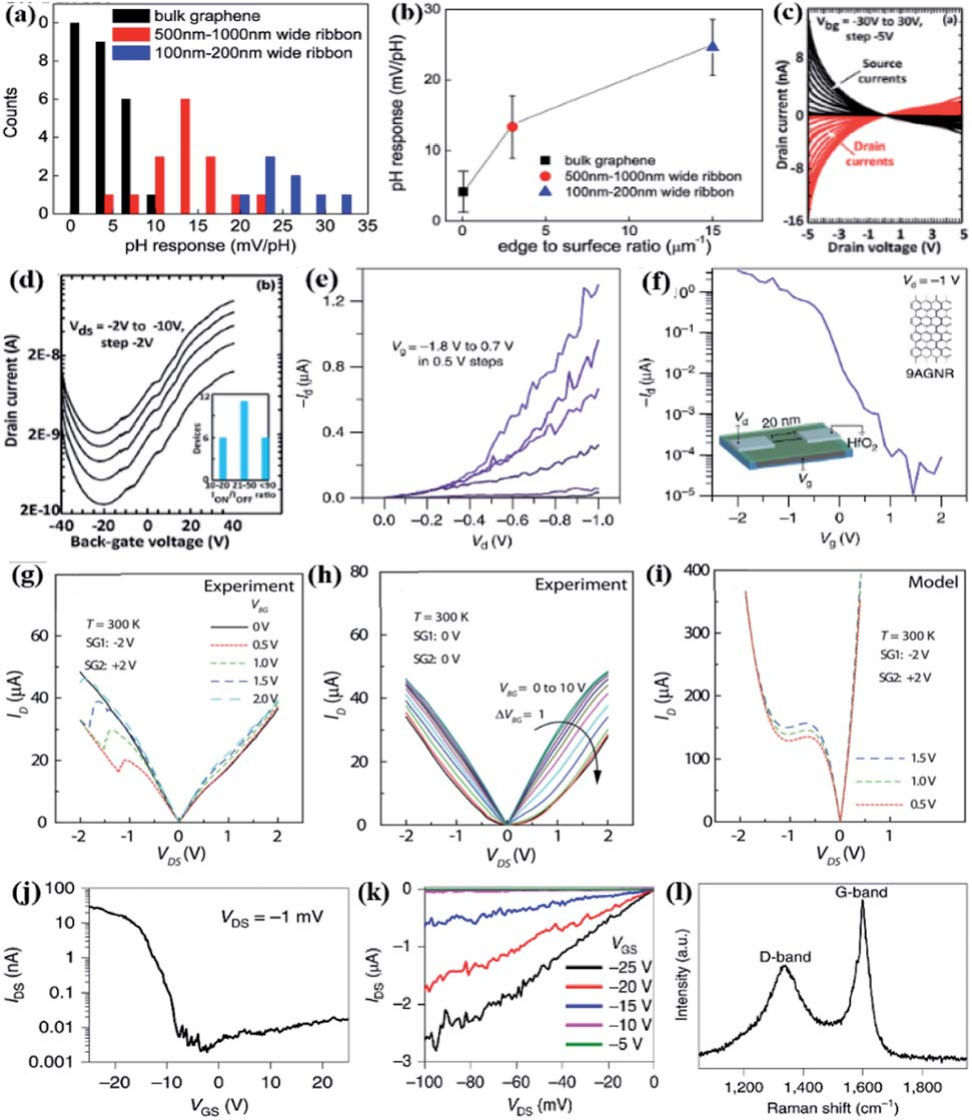
(a) Histogram of the pH response of the device.^185^ (b) Average pH response for different edge ratios.^185^ (c) Output characteristic graph under different back gate bias voltages.^186^ (d) Transmission characteristics of 7-a-GNRs-FET.^186^ (e) 9-a-GNRs-FET: *I*_d_–*V*_g_ characteristics of the scaled.^187^ (f) Graph of *I*_d_–*V*_g_. Inset: Schematic diagram of 9-a-GNRs-FET.^187^ (g) The output characteristic graph of GNRs-FET.^188^ (h) FET behavior in the p-type channel.^188^ (i) Analytical modelling: NDR-FET behavior.^188^ (j) Raman spectrum of GNRs channel in GNRFET.^189^ (k) The output characteristics of GNRFET at room temperature.^189^ (l) Transmission characteristics of GNRFET at room temperature.^189^

In 2013, researchers proved that by introducing edge defects, to achieve a higher pH response of graphene FET, edge defects can be introduced. The decrease in GNR width increases the pH response. In the original graphene FET and 100-nm-wide GNR devices, the lowest sensitivity values (about 0 mV pH^−1^) and highest (30.6 mV pH^−1^) were respectively found (Fig. 8(a)).^185^ When the ratio of side length to the specific surface area increased from 0.04 μm^−1^ to 20 μm^−1^, the pH response increased from 4.2 mV pH^−1^ to 24.6 mV pH^−1^ (Fig. 8(b)).^185^ Obviously, increasing the edge defects can effectively improve the pH response, by reducing the GNR width. Moreover, the change of pH value can cause the reversibility of the Dirac point, which indicates that hydroxide ions can be adsorbed on the edge defects of GNRs through physical methods.

In 2018, Passi and other researchers conducted research on 7-a-GNRs FETs. The atomically accurate and highly aligned 7-a-GNRs can synthesize back-gate transistors using a bottom-up method. The channel length of FETs is approximately 30 times the length of the 30–40 nm nanobelt. Due to the high density of GNRs, it can be assumed that the transmission is much higher than the percolation threshold. The long-channel transistor shows the largest *I*_on_/*I*_off_ current ratio (Fig. 8(c) and (d)). These changes are only part of the definition. The performance of devices with graphical a-GNRs or optimized sources will also be improved, which provides an effective way for the development of GNRs integrated devices and circuits.^186^ Another group of researchers used a high-κ gate dielectric and 9-atom wide GNR channel materials, short channel (about 20 nm) devices were prepared; high-through-current and high *I*_on_/*I*_off_ ∼ 10^5^ FETs were realized at room temperature (Fig. 8(e) and (f)).^187^ If you want to increase the transparency of the barrier, you can increase the gate field near the contact. It is proved that the a-GNRs synthesized from the bottom up are used to successfully fabricate high-performance short channel FETs. Researchers experimentally proved the working of atomic thin GNRs tunneling field-effect transistors (TFETs) at room temperature. The clear and repeatable negative differential resistance (NDR) can be observed; NDR tunnel current density is ∼1 mA μm^−1^ (Fig. 8(g)).^188^ NDR disappears under the positive drain voltage (Fig. 8(h)), and GNR-TFET output characteristics (Fig. 8(i)).^188^ Because of the tunneling of carriers between bands, in the transistor characteristics of the gated GNRs p–n junction, NDR shows repeatability and reversibility. GNR conclusive evidence is adjustable NDR, the bandgap of the lithographically-defined presence and the thinnest Esaki diode. It provides help for the thinnest and scalable development of low-power TFET.

Researchers have fabricated field-effect transistor devices by flattening carbon nanotubes. The field-effect mobility of the device reached 2443 cm^2^ V^−1^ s^−1^, the on-state conductivity was 7.42 mS and the switching ratio reached >10^4^ (Fig. 8(i)–(l)). The band gap of the band is estimated to be ∼0.49 eV. Research data show that, compared with the previous use of graphene nanoribbons or carbon nanotubes to make arrays, the nanoribbons obtained using this method can better meet the needs of integrated circuits. This kind of GNR material with a very small transistor size will have a huge impact on the application of integrated circuits.^189^

In summary, it is found that the bottom-up synthesized GNRs devices are high-performance application devices. The research on the growth mechanism of GNRs has improved the average length of GNRs and the yield of devices. Performance is also improved when edge defects are introduced. With the advantages of low power consumption, stable performance, and strong anti-radiation ability, field-effect devices have gradually replaced triodes in integrated circuits.

### 3.2 Supercapacitor based on GNRs

Between the traditional capacitor and rechargeable battery, there is a new type of energy storage device, the supercapacitor. It not only has the characteristics of fast charging and discharging of capacitors but also the energy storage characteristics of batteries.^190–200^

**Fig. 9:**
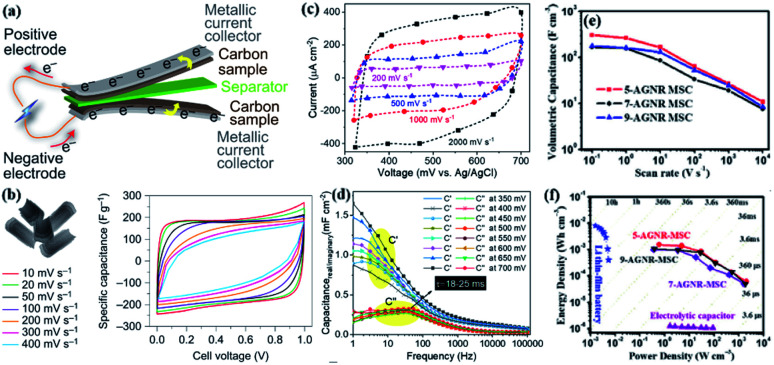
(a) Electrochemical results of GNRs under different bias voltages.^201^ (b) The relationship between cell voltage and specific capacitance at different scanning rates.^201^ (c) Comparison chart of current–voltage characteristics.^202^ (d) Schematic of supercapacitor.^202^ (e) The relationship between volumetric capacitance and scan rate for each width of a-GNRs.^203^ (f) Comparison of power density and energy density of electrolytic capacitors in MSC devices with different widths of a-GNRs.^203^

Some researchers have analyzed the availability of carbon nanorods and GNRs. In the steady-state cyclic voltammogram of GNRs, the regular rectangular shape without any redox peaks shows its excellent capacitance performance (Fig. 9(a)).^201^ As the scan rate increases, the specific capacitance attenuates by 35% (Fig. 9(b)). The outstanding performance of supercapacitors exhibited by such excellent GNRs has progressed the development of two-dimensional carbon materials.^201^ In this way, materials with useful functions can be synthesized. Another group of researchers aimed at the preparation and processing of 1D single-layer GNRs. Electrochemical impedance spectroscopy and cyclic voltammetry show that the average capacitance of 75 μF cm^−2^ at 100 kHz to 1.16 mF cm^−2^ at 1 Hz and 0.35 ± 0.04 mF cm^−2^ (Fig. 9(b) and (c)),^202^ much higher than the upper limit of the capacitance of the carbon-based electrode. Theoretical calculations and experimental results show that by adjusting the structural constraints and doping appropriate functional groups into GNRs at the edges of the reaction, the limitations encountered in charge storage applications can be broken. The high-area capacitance greatly improves the quality control of GNRs, which can solve the newer observation energy storage device and provide help for the study of lateral structural constraints. Klaus of the University of Mainz in Germany used a bottom-up method to synthesize a-GNRs film. This film can be used as an electrode material for micro supercapacitors (MSC) because of its excellent volume capacitance and ultra-high power density. Studies have shown that the electrochemical performance of MSC may be related to the carrier mobility in different GNRs, 5-a-GNRs ≫ 9-a-GNRs > 7-a-GNRs (Fig. 9(e)).^203^ The power density of a-GNRs in MSCs is higher than that of traditional supercapacitors, and more than one order of magnitude higher than aluminum electrolytic capacitors (Fig. 9(f)). The synthesized a-GNRs provide a practical energy storage system for this new one-dimensional carbon material. Such microdevices have ultra-high power density and excellent volumetric capacitance.^203^

The exploration of energy storage in high-speed supercapacitors has always been a hot spot in the study of GNRs. Whether it is physical or chemical methods, precise synthesis will have an impact on the electrochemical properties of GNRs.

### 3.3 Electrocatalyst based on GNRs

**Fig. 10:**
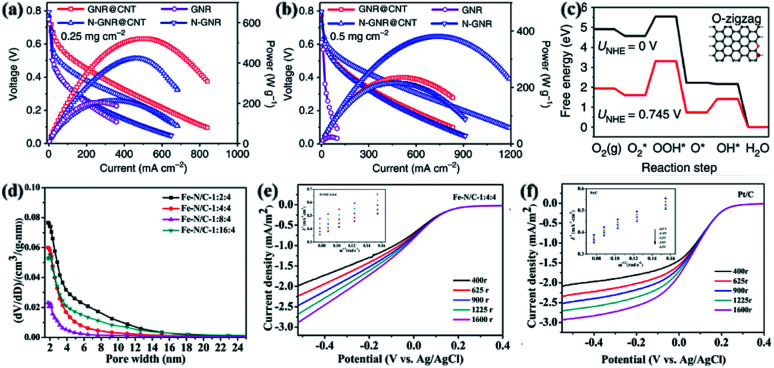
(a) Graphene nanoribbon polarization curves as a function of power density and current density of the surface with cathode catalyst loading of 0.25 mg cm^−2^.^204^ (b) Graphene nanoribbon polarization curves as a function of power density and current density of the surface with cathode catalyst loading of 0.50 mg cm^−2^.^204^ The model of the carbon atom (inset) and the corresponding free energy diagram, (c) carbon atoms at the zigzag edge.^204^ (d) Adsorption–desorption isotherms under different nitrogen-doping levels.^205^ (e) Linear sweep voltammetry (LSV) curve of Fe–N/C at different speeds.^205^ (f) LSV curve of Pt/C at different speeds.^205^

The research team decompressed MWCNTs and synthesized a zigzag edge graphene nanofiber skeleton for oxygen reduction in an electric discharge material exchange membrane fuel cell. Zigzag carbon exhibits peak mass densities of 0.161 W cm^−2^ and 520 W g^−1^ (Fig. 10(a) and (b)),^204^ which is better than most metal electrocatalysts. In an acidic environment, the calculation and experimental results show that the oxygen reduction reaction (ORR) electrocatalytic activity of tortuous carbon atoms is higher than that of oxidizing tortuous carbon and carbon atoms near the cavities (Fig. 10(c)).^204^ The experimental results are the same as the theoretical calculation results, which indicate that this electrocatalyst can be used in inexpensive and good performance proton exchange membrane fuel cells. Due to the higher electrocatalytic activity of the zigzag carbon atoms on ORR, it can play a role in PEMFC applications. It will also be a challenge to synthesize GNRs with jagged edges in the future. Another research team used carbon-based electrocatalysts containing Fe and N elements and graphene nanoribbon@carbon nanotube (Fe–N/GNR@CNT) material as the air-cathode electrocatalyst of the microbial fuel cell (MFC) for the first time. Fe–N/C and industrial Pt/C have similar power generation capacities. Under the condition of different nitrogen doping, the specific surface area of Fe–N/C is also different, indicating that the ORR electrocatalytic activity in the sample is mainly affected by the heteroatom-doping level and the number of active bits (Fig. 10(d)).^205^ The test was carried out in a 50 mM phosphate buffer saline solution (PBS) electrolyte, and the electron transfer number of Fe–N/C was 3.57 (Fig. 10(e)),^205^ The electron transfer number of Pt/C is 3.90 (Fig. 10(f)).^205^ These indicate that the ORR process is mainly dominated by 4 electrons. The presence of Fe, pyridine-N, graphite-N and oxygen-containing groups in GNR@CNT may make the electrocatalyst show good ORR performance. Because of the existence of these doping factors, the electrocatalyst has good ORR performance and its low-cost characteristics, so that GNR@CNT-based materials have become metal catalyst materials to replace other metals.

Precious metal catalysts have always been an obstacle in the field of research.^206–212^ This kind of non-noble metal and metal-free catalyst are ideal materials. The low-cost material will be widely used.

### 3.4 Electrochemical sensor based on GNRs

**Fig. 11:**
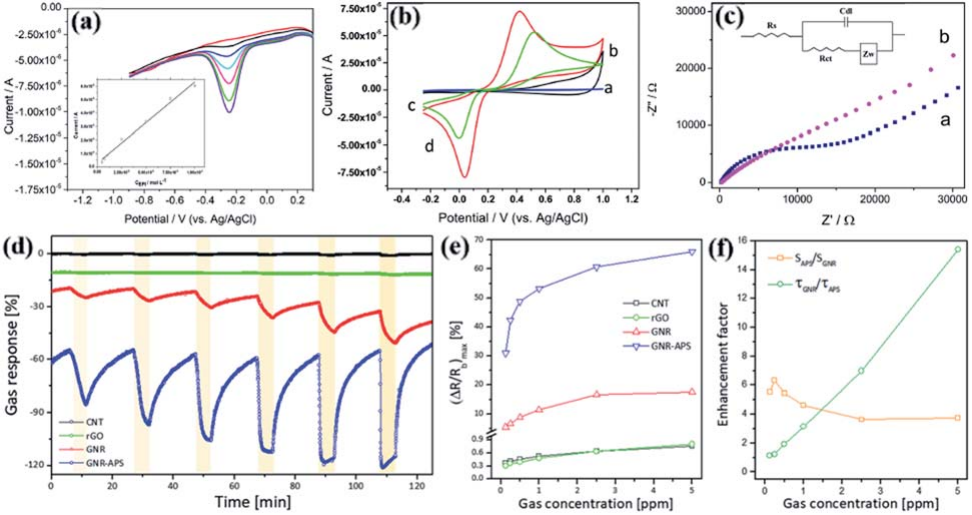
(a) Differential pulse voltammetry (DPV) response of materials in phosphate-buffer solution containing different concentrations of EPI.^213^ (b) Cyclic voltammogram of GCE electrode with/without GNRs modification in 0.1 M PBS at pH = 7.0.^213^ (c) Comparison of electrochemical impedance spectroscopy of GCE electrode with/without GNR-modification, the amplitude is ±10 mV.^213^ (d) The real-time sensing performance of each sensor to various concentrations of NO_2_ from 0.125 to 5 ppm.^214^ (e) The sensor is the maximum resistance change ((Δ*R*/*R*_b_)_max_ (%)) of NO_2_ concentration maximum value.^214^ (f) The relationship between the gas concentration and enhancement factor after APS functionalization.^214^

Casero's research group of the Autonomous University of Madrid synthesized chevron-like GNRs based on solution synthesis, and developed a new type of electrochemical sensor to measure the neurotransmitter epinephrine (EPI). On the sensor surface, electrochemical impedance spectroscopy is used to determine the charge transfer that occurs at the electrode interface. This sensor is used to measure EPI. The reduction peak corresponding to the conversion of adrenaline to white adrenaline is used as the analysis signal (*E* = −0.25 V) instead of the oxidation peak (*E* = +0.6 V) reported in the literature. Reduce interference factors. The electrochemical response of GNRs deposited on glassy carbon electrodes (GCE) sensors to increasing EPI concentration (Fig. 11(a)).^213^ Scientists concluded a linear relationship regarding the modification of electrodes. This shows that when a diffusion-limited transport process is observed, the charge transfer can be significantly improved (Fig. 11(b)).^213^ The research results show that this nanobelt exhibits reliable electrocatalytic activity in the determination of EPI. The linear concentration range is 6.4 × 10^−6^ M to 1.0 × 10^−4^ M (Fig. 11(c)).^213^ By measuring the EPI in the drug sample, its applicability was evaluated, and the result was satisfactory. These results indicate that chemically synthesized GNRs can be successfully applied to the development of electrochemical sensors. The new type of electrochemical sensor synthesized by solution has been well discovered in the laboratory at present, and the follow-up progress is still being strengthened.

Some researchers have studied the application of GNRs in chemical sensors. The chemical modification of aminopropyl silane (APS) molecular edge sites will affect the electrical channel properties of GNRs, thereby improving the sensing performance of GNR sensors. The APS-functionalized GNR sensor has a sensitivity maximal resistance change ((Δ*R*/*R*_b_)_max_) of up to 30% when the concentration of nitrogen dioxide (NO_2_) is 0.125 ppm, and the response time is ultra-fast (about 6 seconds) (Fig. 11(d)).^214^ The response amplitude of each sensor gradually increases with the increase of NO_2_ concentration (Fig. 11(e)).^214^ The enhancement factors of GNRs sensor sensitivity and response time before and after APS functionalization is calculated. When the NO_2_ concentration is higher, the doping effect of APS is better in response time (Fig. 11(f)). Edge-functionalized GNRs have a larger gas response, and GNR-APS sensors also show higher sensitivity, which proves the importance of edge-functionalization of GNRs to chemical sensors.^214^

This sensitive sensor material makes GNRs useful for devices such as transistors. Increasing its conductivity by several orders of magnitude is also beneficial for sensors. Most electrochemical sensors use metal oxide semiconductors as basic materials. Similar sensors include those made of microfluidic biosensors, nanocomposite network membranes and other nanomaterials. These instruments affect all aspects of our lives: medical blood testing, portable photoelectric equipment, food freshness testing, and drug testing in high-risk environments. This kind of portable instrument is easier to carry than the instrument based on GNR material mentioned above. However, some sensors still need long-term research on the production road.^215–226^

### 3.5 Composite material on battery

**Fig. 12:**
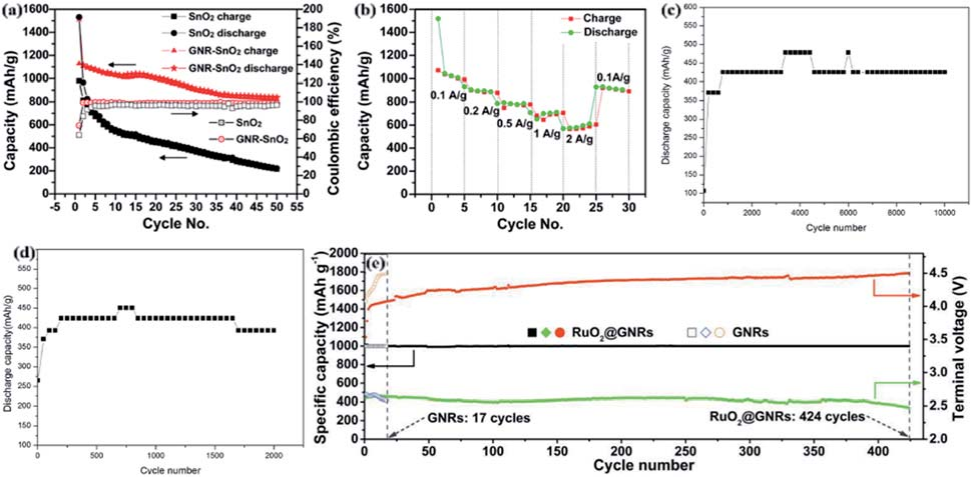
GNRs and SnO_2_ composite materials: (a) the capacity retention and coulombic efficiency at a rate of 100 mA g^−1^.^227^ (b) the ability of composite electrodes of various current densities.^227^ (c) The midpoint discharge capacity of the graphene–ZnO hybrid electrode (38% ZnO).^228^ (d) The midpoint discharge capacity of the graphene–ZnO hybrid electrode (80.7% ZnO).^228^ (e) Comparison of the cycling performance at 200 mA g^−1^.^229^

The research team successfully synthesized high-performance lithium-ion battery composite anodes by evenly distributing SnO_2_ nanoparticles on or in a stack of conductive GNRs. The composite has a high reversible discharge capacity of 1130 mA h g^−1^. In the 50th cycle, when the current density is 0.1 A g^−1^, its discharge capacity remains at about 825 mA h g^−1^ (Fig. 12(a)).^227^ At a current density of 2 A g^−1^, the specific capacity of the composite was 580 mA h g^−1^ (Fig. 12(b)),^227^ which is much higher than the theoretical capacity of graphite. The high-capacity SnO_2_ composite material has helped the development path of lithium storage based on GNRs-based composite materials and other metal oxides. However, there are still certain difficulties in the preparation and synthesis. Cui's research team designed graphene–ZnO composite electrodes, which can be used in nickel–zinc secondary batteries. They cut graphene into short nanoribbons *in situ*, effectively fixing a large number of zinc atoms on its surface. In the test cycle, the mass ratio of graphene to zinc oxide is 31 : 19 (38% ZnO). The electrochemical performance is very stable, and the cycle life of the battery is far greater than 10 000 times (Fig. 12(c)).^228^ When the mass ratio of graphene to zinc oxide is 80.7% and the number of cycles is greater than 1700, the midpoint discharge capacity decreases slightly, indicating that the cycle stability is weaker than that of 38% ZnO (Fig. 12(d)). For traditional Ni–Zn batteries, the newly designed Ni–Zn batteries have excellent discharge capacity and easy-to-operate preparation methods. This superior electrochemical performance is very useful in the electrochemical field.^228^

Xu's research team has discovered a new type of cathode with GNRs decorated with RuO_2_ catalysts by decompressing MWCNTs. The decorative RuO_2_ catalyst was obtained by decompressing MWCNTs. RuO_2_ introduced by a simple drip method enhanced the kinetics of oxygen evolution reaction and significantly reduced the charge overpotential. More importantly, the lithium–oxygen battery with RuO_2_ modified graphene nanoribbon cathode has good 424 cycle stability at a capacity of 1000 mA h g^−1^ (Fig. 12(e)).^229^ Due to the different substrate structures, Li_2_–O_2_ deposited on GNRs and Li_2_–O_2_ deposited on MWCNTs have completely different surface morphologies. This provides development assistance for the high-efficiency air electrode design of high-performance lithium–oxygen batteries. It will also provide help in subsequent research on battery energy efficiency and cycle life.

The research on GNRs composite materials has provided an effective design for traditional batteries, both the cycle life and discharge capacity have been significantly improved. Researchers will continue to explore their characteristics.^230–234^

## 4 Conclusion

GNRs exhibit special electrical properties through their unique electronic structures. Researchers have found that GNRs themselves have atomic-level precision and stability. When the width, boundaries, defects, stress, surface adsorption, doping of other atoms, *etc.* of GNRs are adjusted, slight changes will cause changes in the electronic structure and electrical properties of GNRs.

In this work, we have summarized the latest research progress on the electrical properties of GNRs and the application prospects of GNRs. The electrical properties of GNRs: metallic, topological, transport, conductivity, acoustoelectric effect, electron emission, electrochemistry, electrocatalytic activity, *etc.* are summarized. GNRs not only have broad prospects in the field of electricity, but also have outstanding research in the fields of optics, heat, and mechanics.

We have also obtained GNRs as basic materials suitable for FETs, electrochemical sensors, supercapacitors, electrocatalysts, and other electronic devices. The application of GNRs to a variety of electronic devices broadened the ideas and methods. For example, GNRs are suitable for the production of high-performance electronic products as a substitute for silicon; the edge effect of GNRs can be controlled to change the metallicity of devices; GNRs have good conductivity and can produce large-scale, cheap and high-conductivity integrated circuit devices; GNRs that are used as the composite material in the battery have superior energy conversion efficiency and so on. When exploring the width-dependent semiconductor band gap driven by the quantum confinement effect, the closed state in electronics is realized, which contributes to the study of quantum confinement, and at the same time provides substantial help in the follow-up low-power electronic technology. GNRs have a large battery capacity and long charging time, which solves the shortage of energy batteries in the past and paves the way for the future application of the new energy battery industry. Especially, supercapacitors based on GNRs, GNRs have excellent conductivity and high specific surface area performance. With the development of industry and productivity, high-precision supercapacitors are prepared to meet actual needs. For example, high power density, long cycle life, low and high-temperature resistance, higher voltage, high safety supercapacitors can be used in good energy storage devices, such as truck starting, windmills, and electric meters.

Compared with traditional research, in recent years, researchers have devoted themselves to creating new materials. The synthesis is limited to small fragments, and a large number of production methods are yet to be explored. The research in the field has great potential, especially related to electrical characteristics. GNRs have bright application prospects in many fields such as electronic devices.

## Author contributions

The content and design: Jingang Wang and Yi Zou; writing manuscripts: Jingang Wang; original writing: Wenjing Bo.

## Conflicts of interest

There are no conflicts to declare.

## Supplementary Material
